# The Awesome Power of Yeast Evolutionary Genetics: New Genome Sequences and Strain Resources for the *Saccharomyces sensu stricto* Genus

**DOI:** 10.1534/g3.111.000273

**Published:** 2011-06-01

**Authors:** Devin R. Scannell, Oliver A. Zill, Antonis Rokas, Celia Payen, Maitreya J. Dunham, Michael B. Eisen, Jasper Rine, Mark Johnston, Chris Todd Hittinger

**Affiliations:** * Department of Molecular and Cell Biology and California Institute for Quantitative Biosciences, UC Berkeley, Berkeley, California 94720-3220; † Department of Biological Sciences, Vanderbilt University, Nashville, Tennessee 37235; ‡ Department of Genome Sciences, University of Washington, Seattle, Washington 98195; § Howard Hughes Medical Institute, University of California, Berkeley, Berkeley, California 94720; ** Department of Biochemistry and Molecular Genetics, University of Colorado School of Medicine, Aurora, Colorado 80045-2530; †† Center for Genome Sciences, Department of Genetics, Washington University in St. Louis School of Medicine, St. Louis, Missouri 63108-2212

**Keywords:** *Saccharomyces* genome, genome assembly, evolutionary genetics, *sensu stricto*, genomics, yeast species

## Abstract

High-quality, well-annotated genome sequences and standardized laboratory strains fuel experimental and evolutionary research. We present improved genome sequences of three species of *Saccharomyces sensu stricto* yeasts: *S. bayanus* var. *uvarum* (CBS 7001), *S. kudriavzevii* (IFO 1802^T^ and ZP 591), and *S. mikatae* (IFO 1815^T^), and describe their comparison to the genomes of *S. cerevisiae* and *S. paradoxus*. The new sequences, derived by assembling millions of short DNA sequence reads together with previously published Sanger shotgun reads, have vastly greater long-range continuity and far fewer gaps than the previously available genome sequences. New gene predictions defined a set of 5261 protein-coding orthologs across the five most commonly studied *Saccharomyces* yeasts, enabling a re-examination of the tempo and mode of yeast gene evolution and improved inferences of species-specific gains and losses. To facilitate experimental investigations, we generated genetically marked, stable haploid strains for all three of these *Saccharomyces* species. These nearly complete genome sequences and the collection of genetically marked strains provide a valuable toolset for comparative studies of gene function, metabolism, and evolution, and render *Saccharomyces sensu stricto* the most experimentally tractable model genus. These resources are freely available and accessible through www.SaccharomycesSensuStricto.org.

Hemiascomycete yeasts (subphylum Saccharomycotina) have emerged as a preeminent phylogenetic clade for comparative genomics due to their small, streamlined genomes, a wealth of functional data, and genetic diversity spanning 500–1000 million years of evolution (Dujon 2010; Dujon *et al.* 2004; Piskur and Langkjaer 2004; Taylor and Berbee 2006). Although low-to-medium-coverage genome sequences of many species in this group have been determined (Scannell *et al.* 2007a), relatively few are complete and well-annotated (Dujon 2010). Most studies have focused on large-scale evolutionary changes, such as the whole-genome duplication that occurred within the *Saccharomyces* complex of species (Wolfe and Shields 1997; Dietrich *et al.* 2004; Kellis *et al.* 2004; Scannell *et al.* 2006; Scannell *et al.* 2007b; Wapinski *et al.* 2007b). Broad comparative analyses have been critical to our understanding of how genomes evolve over long time scales, and for describing what makes fungi distinct from plants and animals. Determining the genetic bases for more recent and rapid evolutionary changes within and between species remains an area of active research across many phyla (Atwell *et al.* 2010; Peichel 2005; Prud'homme *et al.* 2007; Seidel *et al.* 2008), for which unfinished genome sequences have proven inadequate.

Comparative genomic analyses of entire genera greatly facilitate evolutionary research, but few genera have the resources—both genetic and genomic—required to support such work (Clark *et al.* 2007; Butler *et al.* 2009). Smaller-scale comparative studies in yeast have already provided mechanistic insights into key evolutionary concepts, such as speciation (Chou *et al.* 2010; Greig *et al.* 2002; Lee *et al.* 2008; Greig 2009), life history variation (Gerke *et al.* 2009), *cis*-regulatory evolution (Fay and Benavides 2005; Fidalgo *et al.* 2006), conditional-fitness tradeoffs (Will *et al.* 2010), and the long-term maintenance of complex genetic variation (Hittinger *et al.* 2010). Further, comparative analyses of species closely related to a classical model organism can reveal regulatory pathways not readily discoverable in a single “model” species (Zill and Rine 2008), provided genetic tools exist in the “nonmodel” species. Next-generation genomics technologies make the sequencing of entire genera labor- and cost-efficient, bridging the gulf between research on an established model organism and comparative research on its relatives. Endowing several con-generic species with the genetic prowess of their classical model relative would revolutionize the study of the genetic basis of evolution by allowing reciprocal experiments across a model genus.

Although the genome sequences of several species within multiple eukaryotic genera have been determined (*e.g.*, Stein *et al.* 2003; Clark *et al.* 2007; Rokas *et al.* 2007; Butler *et al.* 2009), none of these are amenable to nucleotide-level targeted reciprocal genetic analyses between a classical model organism and multiple close relatives. The *Saccharomyces sensu stricto* genus, which includes the model organism *S. cerevisiae*, offers a unique opportunity. This clade includes at least five other natural species—*S. paradoxus*, *S. mikatae*, *S. arboricolus*, *S. kudriavzevii*, and *S. bayanus*—and one hybrid species—, *S. pastorianus* (Figure 1A) (Naumov *et al.* 2000; Wang and Bai 2008; Nakao *et al.* 2009). (We note that a recent study provides strong evidence that *S. bayanus* var. *bayanus* and *S. bayanus* var. *uvarum* are genetically and ecologically isolated sister species from two distinct lineages (Libkind, Hittinger, *et al.*, unpublished data). The genomics and genetics communities have used *S. bayanus* to refer to *S. bayanus* var. *uvarum*, and we continue that convention here.) The *Saccharomyces sensu stricto* genus is thought to have evolved ∼20 million years ago, and its species have a level of nucleotide divergence similar to that found between birds and humans (Dujon 2006). However, because yeasts lack a fossil record, the estimation of absolute divergence times for any set of yeast species is imprecise (Taylor and Berbee 2006).

**Figure 1:**
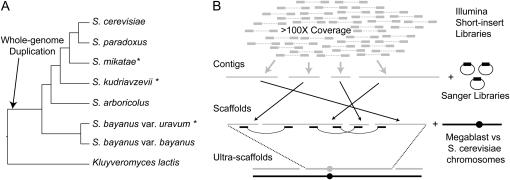
Resequencing and assembling the genomes of three *Saccharomyces* species. (A) Schematic showing phylogenetic relationships among nonhybrid members of the *Saccharomyces sensu stricto* genus plus the outgroup *Kluyveromyces lactis* based on (Kurtzman and Robnett 2003), (Nieduszynski and Liti 2011), and (Libkind, Hittinger *et al.*, unpublished data). Branch lengths are not proportional to sequence divergence. The branch on which the whole-genome duplication occurred is marked. (B) Schematic depicting co-assembly of genomes from Illumina short-insert paired-end reads and mate-pair Sanger shotgun reads. Illumina reads were used to build contigs, which were stitched into scaffolds using mate-pair reads from the longer-insert Sanger libraries. Scaffolds were then joined into ultra-scaffolds (contiguous with chromosomes) using MEGABLAST and manual scaffold ordering.

The genomes of *S. paradoxus*, *S. mikatae*, *S. kudriavzevii*, and *S. bayanus* were originally sequenced to low-medium coverage (3-8×) (Cliften *et al.* 2003; Kellis *et al.* 2003). These sequence assemblies were far from complete with N50 values (*i.e.*, the minimum contig/scaffold length above which 50% of the entire assembly is contained in contigs or scaffolds equal to or larger than this value) well below 100 kb (as low as 11 kb for *S. kudriavzevii*). Due to the large number of gaps in each genome sequence, fewer than half of the potential orthologs of *S. cerevisiae* genes (2742/6615) were fully assembled and annotated across all four con-generic species. The missing data have both limited comprehensive sequence-based evolutionary analyses, and forced individual investigators to perform targeted resequencing to support sequence and genetic analyses of specific genes (Guan *et al.* 2010; Zill *et al.* 2010; Hittinger *et al.* 2004; Airoldi *et al.* 2009; Gallagher *et al.* 2009).

To facilitate evolutionary genetic and genomic analyses within the *Saccharomyces sensu stricto* genus, we resequenced to high coverage and reassembled the genome sequences of *S. mikatae*, *S. kudriavzevii*, and *S. bayanus*. With these new genome sequences, an improved assembly of the *S. paradoxus* genome (Liti *et al.* 2009), and the reference genome of *S. cerevisiae* (Goffeau *et al.* 1996), we determined the average and branch-specific evolutionary rates for a revised set of 5261 complete, annotated protein-coding orthologs across five *Saccharomyces* species, and identified 123 genes that may have been targets of positive selection. Through a relaxed-clock phylogenetic analysis, we obtained more accurate and precise relative estimates of interspecies divergence. Finally, we derived marked laboratory strains of the three species, permitting comparative genetic experiments at an unprecedented level of phylogenetic resolution and power within the *Saccharomyces* genus.

## Materials and Methods

### Genome sequencing

Paired-end Illumina sequencing libraries were prepared from sonicated or nebulized genomic DNA according to manufacturer protocols with certain modifications (Hittinger *et al.* 2010; Lefrancois *et al.* 2009). For *S. bayanus*, a *MAT***a** *hoΔ::NatMX* derivative of CBS 7001 was sequenced. For *S. mikatae*, sheared DNA isolated from strain IFO 1815^T^ was processed by an IntegenX robot. For *S. kudriavzevii*, haploid derivatives FM1097 and FM1109 were sequenced. Mean insert sizes (±SD) of Illumina libraries, as determined by SOAPdenovo, were as follows: IFO 1815^T^, 259 bp (±76 bp); IFO 1802^T^, 203 bp (±20 bp); ZP 591, 226 bp (±23 bp); CBS 7001, 437 bp (±45 bp).

Sequencing was performed on Illumina Genome Analyzer II or II_x_ machines at the Vincent Coates Genome Sequencing Lab, QB3, Berkeley, CA and at the University of Colorado School of Medicine. Read lengths varied for each strain as follows: *S. bayanus*, 51 bases; *S. mikatae*, 80 bases; *S. kudriavzevii*, 114 bases. All raw read data have been deposited in the SRA at NCBI (http://ncbi.nlm.nih.gov/sra) in SRP006340 of SRA034902. Reads, assemblies, and annotation files are freely available at http://www.SaccharomycesSensuStricto.org.

### Co-assembly of Illumina and Sanger reads

Sanger reads were quality trimmed using LUCY (Chou and Holmes 2001) (default parameters, except −minimum 60). Vector sequences at the 5′ end of reads were masked using Figaro (White *et al.* 2008) (default parameters). Reads where more than 20% of bases were determined to be of vector origin were discarded; all others were 5′ trimmed and retained. Reads with remaining significant homology to the NCBI UniVec database (downloaded June 12, 2009) detected by Crossmatch (Ewing and Green 1998) (default parameters) were discarded. Reads shorter than 60 bp and unpaired reads were discarded. Reads were 3′ trimmed to a maximum of 180 bp.

Illumina reads were quality trimmed using fsq2fsa (available from D. Scannell on request), which trims bases from the 3′ ends of reads based on the Illumina quality score in small windows. The quality score was optimized for each dataset by assembling all reads with SOAPdenovo (http://soap.genomics.org.cn/soapdenovo.html; Version 1.05; July 29, 2010) (default params; −K = 31) and selecting the assembly with the best N50 (other metrics produced similar results). In addition, we used fsq2fsa to eliminate reads with significant matches to Illumina adapters and used the SOAP Corrector (default parameters) tool to correct errors in reads. We did not hard trim bases from the 5′ ends of reads because doing so did not improve assemblies.

Assemblies were generated using SOAPdenovo (default parameters except −K as described below) using Illumina reads for both contig generation and scaffolding (rank = 1, pair_num_cutoff = 3, asm_flags = 3, map_len = 32) and Sanger reads for scaffolding only (rank = 2, pair_num_cutoff = 3, asm_flags = 2, map_len = 32). Sanger libraries of different origins were supplied to SOAPdenovo separately and the insert sizes for each determined by BLASTing against contigs longer than 10 kb. We optimized the −K parameter (Kmer) separately for each assembly by examining a range of values in the range 17–61. Finally, we used the SOAP GapCloser tool (default parameters) to fill assembly gaps using Illumina reads only.

### Genome annotation

Our new assemblies as well as previously published sequences for *S. cerevisiae* (Engel *et al.* 2010; Goffeau *et al.* 1996) and *S. paradoxus* (Liti *et al.* 2009) were all annotated with a common pipeline. We used HMMER 1.8.4 (http://hmmer.janelia.org/software/archive) to detect high scoring matches to an HMM created from an alignment of *S. cerevisiae* introns and flanking sequences. We then generated all ORFs above a context-dependent minimum size (60 bp at contigs ends and intron splice-sites, 300 bp at other locations), combined these with the putative splice-sites and selected the most upstream ATG as the putative start codon. Initially, we retained only possible gene models longer than 300 bp. Shorter genes were later recovered by extending BLAST HSPs (described below). Following this procedure, potential ORFs that spanned high-scoring matches to the splice site model were represented by multiple alternative gene models. At each locus, we aligned alternative models to the best available homolog (typically from *S. cerevisiae*) using Exonerate (Slater and Birney 2005) (−model affine:global −exhaustive 1 −bestn 1) and selected the model with the highest score. We sought positive evidence for all remaining gene models by BLASTing (Altschul *et al.* 1997) against a local database of yeast proteins assembled from completely sequenced yeast genomes (Wood *et al.* 2002; Cliften *et al.* 2003; Dujon *et al.* 2004; Kellis *et al.* 2004; Souciet *et al.* 2009), the NCBI nr protein database, and *S. cerevisiae* Ty and LTR sequences downloaded from *Saccharomyces* Genome Database (Engel *et al.* 2010) (SGD, http://www.yeastgenome.org). In addition, we used HMMER3 (Eddy 2009) to search our gene models against a database of 4704 protein HMMs derived from the Yeast Gene Order Browser (Byrne and Wolfe 2006) (YGOB, http://wolfe.gen.tcd.ie/ygob). In order to recover small genes or rapidly evolving genes, we subjected all presumed intergenic regions to low stringency BLAST searches against our local database of yeast proteins and searched genomic regions predicted by synteny to contain YGOB matches with our YGOB-HMM library. To detect potential novel genes, we used linear discriminant analysis to assign ORFs lacking homology to genes in the YGOB or yeast ortholog databases a “coding probability” based on whether their codon usage frequencies more closely resembled confirmed protein-coding genes or confirmed intergenic regions. Finally, we eliminated poorly supported gene models that overlapped well-supported models and merged neighboring models that were likely to be fragments of the same gene. The fragmented gene models were typically created by scaffold gaps or frameshifts (either real or due to sequencing errors). The remaining models were stratified according to the quality of the supporting evidence and comprised our initial protein-coding gene set. We used tRNA-ScanSE to identify tRNA genes (Lowe and Eddy 1997).

As described in more detail below (*Ortholog Detection and Assignment*), we identified putative orthologs among the genomes of five species’ “representative” strains (*i.e.*, *S. mikatae* IFO 1815^T^, *S. kudriavzevii* IFO 1802^T^, *S. bayanus* CBS 7001, *S. cerevisiae* S288C, and *S. paradoxus* CBS 432^T^). We used interspecies comparisons to improve annotations for these five strains in two ways. First, where intron–exon structures differed among species, we used Exonerate (−model protein2genome −exhaustive 0 −bestn 1) to perform a multiframe alignment of the closest homologous protein to the genomic region around each gene. Resulting models were preferred if they improved the consistency among species and typically contained small (2-7 bp) first exons or multiple frameshifts. Second, we aligned the putative 5′ termini and upstream regions of genes and selected the start codon that minimized the variation among species while maximizing overall gene length.

### Ortholog detection and assignment

We detected single-copy orthologs among the five strains using a two-step procedure that incorporates both homology and synteny at both steps. First, we grouped genes across all species into families according to the best match in our YGOB-HMM database. Within each family, we selected the species with most representatives and founded an orthology group (or “orthogroup”) with each copy. We then assigned genes from other species to orthogroups if they exceeded the specified minimum level of synteny support and the level of support for the next best orthogroup was significantly less. To compose a synteny statistic, we counted the number of YGOB-HMM families that were shared between a ten-gene window centered on the query gene and one centered on the focal orthogroup. We computed the hypergeometric probability of this observation assuming a total genome-size of 4704 genes (the number of YGOB models that can be distinguished) and used −log_10_(P_hyper_) as our synteny statistic. Once all possible genes in a family were assigned to orthogroups, five-membered orthogroups were considered complete and removed. Remaining family members were then assigned to the remaining incomplete orthogroups and the procedure iterated until steady state.

Once our initial set of orthogroups was defined we looked in the genomic regions between orthogroups for additional sets of syntenic orthologs that were not related to any of the YGOB-HMM families or had been missed for lack of synteny or other reasons. If orthologs were present at a genomic location in only a subset of species, we reannotated the syntenic region in the un-represented species to recover any missed orthologs. Finally, we re-examined all orthogroups and rejected those that exhibited either weak synteny or weak homology support. Orthologous genes were aligned in protein-space with FSA (Bradley *et al.* 2009) and back-translated to DNA using RevTrans (Wernersson and Pedersen 2003). A comparison of gene structures and lengths suggested at least 4792 (∼87%) of our orthologous gene sets were of high quality (Table S1).

### Evolutionary analyses

To assess how improved genome sequences facilitated thorough evolutionary analyses, we determined the number of the single-copy orthologs present in the original 2003 genome annotations, which were not published jointly (Kellis *et al.* 2003, Cliften *et al.* 2003). We considered the more complete Kellis *et al.* 2003 annotations for *S. bayanus*, *S. paradoxus*, and *S. mikatae*; the Cliften *et al.* 2003 annotations for *S. kudriavzevii*; and the *S. cerevisiae* annotations as downloaded from SGD on February 13, 2008. Annotations were taken to designate orthology if the original authors used the systematic name for *S. cerevisiae* gene along with the systematic name for the species in question in the versions deposited in SGD. As before, this analysis yielded 2805 genes with orthologs previously designated in all five *Saccharomyces sensu stricto* species (Hittinger *et al.* 2010). However, the Kellis *et al.* nomenclature allowed multiple genes to be named for the same *S. cerevisiae* gene (*e.g.*, lineage-specific duplicates that are all orthologous to a single *S. cerevisiae* gene). Excluding these genes, only 2742 genes were actually annotated as single-copy (1:1:1:1:1) orthologs in the 2003 genomes.

To construct a data matrix for codon-based analyses, we first filtered out all ortholog groups from the new assemblies that did not contain orthologs from all five species as well as any ortholog groups in which more than 75% of alignment columns had missing data or gaps. The resulting data matrix was composed of 5152 orthologs and contained 7,880,523 nucleotide columns (the average length of a coding sequence alignment was ∼1530 base pairs).

**Figure 2:**
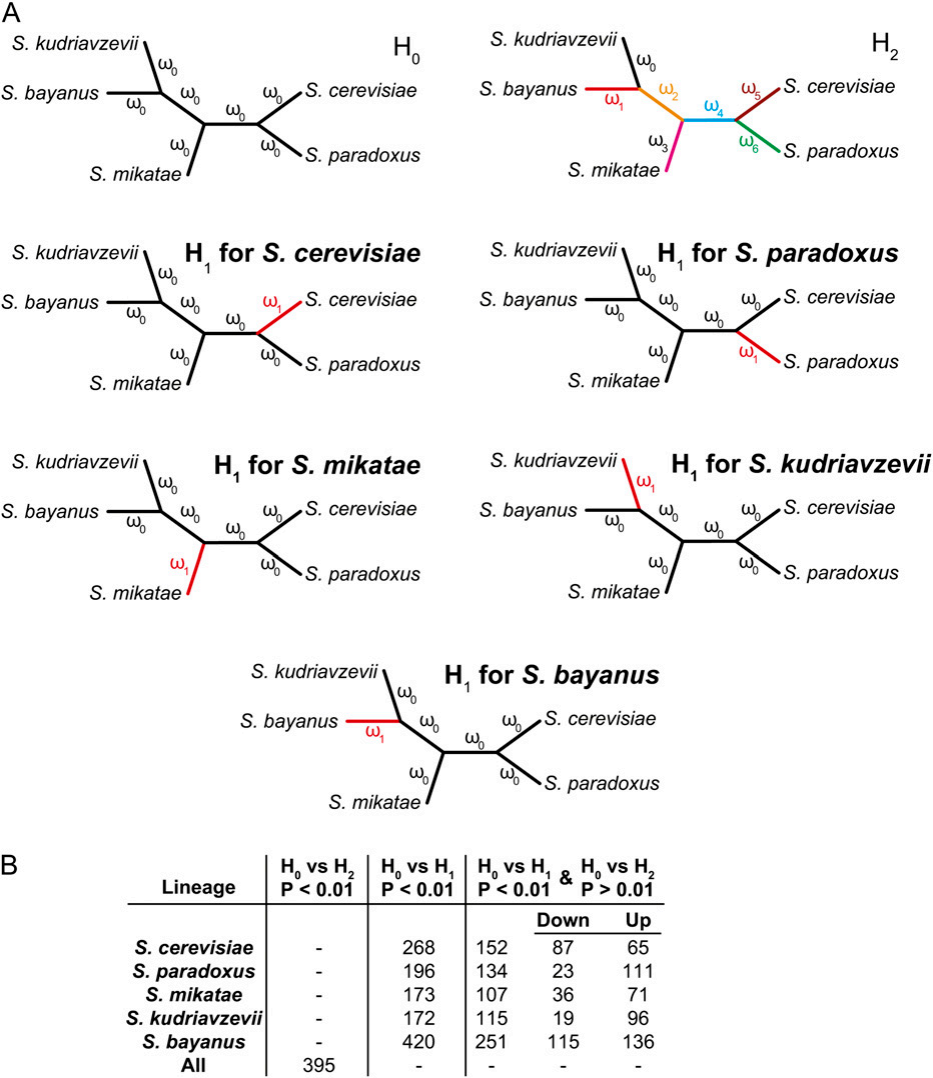
Genes exhibiting lineage-specific rates of evolution in the *Saccharomyces sensu stricto* genus. (A) The three alternative hypotheses designed to test whether genes are evolving at a different rate in each of five species of the *Saccharomyces sensu stricto* genus. Under hypothesis H_0_ all branches of the tree exhibit the same ω ratio of nonsynonymous to synonymous substitutions. Under the set of H_1_ hypotheses, the ω ratio along a given species’ branch is different from that along all other branches of the tree. Under the H_2_ hypothesis, each branch exhibits its own ω ratio. (B) Numbers of genes with lineage-specific rates of evolution in the *Saccharomyces sensu stricto* genus.

We examined variation in selection pressure along branches of the species tree and tested each gene for evidence of positive selection using the CODEML module from PAML (Yang 2007). To examine selection pressure variation along branches of the species tree for each gene, we evaluated the log likelihood of two alternative hypotheses relative to the null hypothesis H_0_, under which all branches of the phylogeny exhibited the same ω ratio of nonsynonymous (dN) to synonymous (dS) substitutions (Figure 2A). The first set of alternative hypotheses (H_1_) stated that the ω ratio along the external branch for a given species was different from that in the rest of the branches of the phylogeny (Figure 2A). To discriminate between genes that were consistent with a different ω ratio only along a particular species from genes that were consistent with distinct ω ratios in all branches of the phylogeny, we also tested the second alternative hypothesis (H_2_), in which each lineage exhibited its own ω ratio against the H_0_ hypothesis (Figure 2A). To test for positive selection in each gene, we first evaluated the log likelihood of the null M7 model. Under M7, ω values at different codon positions in a gene follow a beta distribution, where ω is constrained to fall between zero and one. We then compared the log likelihood of the M7 model relative to that estimated by the alternative model M8, which, in addition to the zero to one beta distribution for ω values, also allows for a subset of codon sites to have ω values above one. We excluded all genes with dS values of zero. All tests were done at *P* = 0.01 significance. In File S1, we have also provided an optional filter to remove genes from these screens whose high ω values were driven by abnormally low denominators (*i.e.*, dS values two standard deviations below the mean). Depending on the specific alternative hypothesis, application of the filter removes ∼5–10% of the genes rejecting H_0_, including many genes encoding ribosomal proteins and other translation factors that are not likely to be experiencing lineage-specific selection.

We inferred relative divergence times for the yeast phylogeny using the BEAST software, version 1.6.1 (Drummond and Rambaut 2007) on a data set of 106 genes spanning the yeast genome (Rokas *et al.* 2003). Because the fungal and yeast fossil records are sparse and reliable fossil calibration points unavailable, we estimated all branches in units of substitution/site. For all phylogenetic analyses using BEAST, we assumed the SRD06 model of sequence evolution (Shapiro *et al.* 2006), allowing for rate heterogeneity across sites through the gamma distribution, and the uncorrelated log-normal relaxed clock model. We chose the Yule process as our tree prior. We ran three independent runs for 10,000,000 generations. We verified the convergence of runs by examining the effective sample size of the likelihood and posterior probability parameters for each analysis (>100 parameters) and by visually inspecting the likelihood and posterior probability distributions across independent runs. We discarded the first 10% of sampled data points from each run as burn-in.

### Identifying potential gene gains and losses

We used a variety of approaches to identify gene gain and loss candidates that we subsequently manually inspected. First, we took a synteny-based approach to identify genes that were lost or gained in internal chromosomal regions with well-conserved synteny. Briefly, we “walked” along the genome of *S. cerevisiae* and checked the region between each orthogroup (conserved 1:1:1:1:1 syntenic orthologs) and the previous orthogroup for the presence of genes in one or more species. We excluded regions that contained assembly gaps in any of the five species or where there were more than three intervening features in any species. As these criteria were restrictive, we also took a homology-based approach, grouping genes by their homology to YGOB-HMM families and selecting families that differed in size among species (but excluding the small number of very large families). In addition, we examined any genes that had evidence of function (see *Genome annotation*) but which had no detectable homology to either a YGOB-HMM or to a gene in SGD. Finally, we also sought to identify cases where although a gene was detectable by homology, the reading-frame had been disabled. To do this, we sorted genes by the number of frameshifts required to reconstitute a full-length gene during the annotation process and examined any gene with three or more disruptions. We also sorted orthogroups by the standard deviation of gene length divided by the mean gene length and examined the top 200 outliers to detect genes that had been severely truncated.

### Strain construction

Prototrophic diploid yeast strains CBS 7001, IFO 1802^T^, ZP 591, and IFO 1815^T^ were made heterothallic by inactivating the *HO* gene. *HO*/*hoΔ* heterozygous diploids were sporulated and tetrads were dissected to isolate *MAT***a** and *MAT*α *hoΔ* haploids. For *S. mikatae* and *S. kudriavzevii*, auxotrophic markers were generated by gene targeting. For *S. bayanus*, auxotrophic markers were introduced by EMS mutagenesis as described previously (Zill and Rine 2008). To generate the *S. kudriavzevii trp1Δ0* and *ura3Δ0 delitto perfetto* alleles, prototrophic strains were transformed with DNA oligos and/or PCR products encoding a direct junction of the sequences immediately upstream of the start codon and downstream of the stop codon (Storici *et al.* 2001). Transformed pools were grown on 5-FAA and 5-FOA media to select for strains that lack functional *TRP1* and *URA3*, respectively. All other homologous-recombination-based gene targeting was performed by one-step gene replacement using standard drug-resistance cassettes (Guldener *et al.* 1996; Longtine *et al.* 1998; Goldstein and Mccusker 1999) or standard two-step replacements using *URA3* (Storici and Resnick 2006). All gene disruptions were confirmed using PCR and/or sequencing to examine the 5′ and 3′ ends of targeted ORFs.

Transformation protocols for *S. bayanus* (Zill and Rine 2008; Gallagher *et al.* 2009) and *S. kudriavzevii* (Hittinger *et al.* 2010) have been described. Here we again summarize the relevant modifications to the standard PEG/LiAc heat-shock protocol used for *S. cerevisiae* transformation. All of the non–*S. cerevisiae* species appear to be quite sensitive to prolonged heat shock at 42°C. For *S. bayanus*, heat shock was performed for five minutes at 42°C after a 10-minute incubation at room temperature in the transformation mixture. For *S. kudriavzevii*, heat shock was performed for 30 min at 34°C. For *S. mikatae*, heat shock was performed for five minutes at 37°C after a 10-minute incubation at room temperature. For all three species, subsequent outgrowth and culture were performed at room temperature (∼23°C). For *S. bayanus*, gene disruption primers contained 40nt homologous to the sequences immediately flanking the targeted ORF. For *S. mikatae* and *S. kudriavzevii* the primers had 50 nt and 70 nt of homology, respectively.

## Results

### Improved genome assemblies for *S. mikatae*, *S. kudriavzevii*, and *S. bayanus*

The taxonomic type or other representative strains of *S. mikatae*, *S. kudriavzevii*, and *S. bayanus* were previously sequenced to 3-8× coverage with Sanger sequencing technology (Cliften *et al.* 2003; Kellis *et al.* 2003). Though this depth of sequencing provided value in terms of genome coverage, and where coverage was highest provided good long-range continuity (Cliften *et al.* 2006), the resulting assemblies had many gaps and a moderate number of errors. To obtain more complete assemblies that would support base-pair level analyses of these three species, we sequenced short-insert (203-437 bp) Illumina paired-end libraries to greater than 100× coverage (Table 1) and assembled these together with the available sequences (Figure 1B). The high raw coverage afforded by the short-read technology minimized erroneous base calls and gaps in unique regions, whereas the longer inserts from the shotgun sequencing projects (3198-4789 bp inserts; trimmed reads averaged 179 bp in length) helped bridge repetitive regions and establish long-range scaffolds. In addition, we sequenced and assembled a genetically and phenotypically diverse *S. kudriavzevii* strain (ZP 591) (Sampaio and Goncalves 2008) from Illumina reads only, as there were no Sanger shotgun reads available.

**Table 1:** Short-read library statistics

	Library Insert (bp)	Read Length (bp)	Assembly Kmer	Fold Coverage		
				Raw	Processed*a*	Kmer
*S. bayanus* (CBS 7001)	437	51	31	140.7	109.5	45.1
*S. kudriavzevii* (IFO 1802^T^)	203	114	61	272.2	202.5	95.9
*S. kudriavzevii* (ZP 591)	223	114	61	269.4	207.8	98.4
*S. mikatae* (IFO 1815^T^)	259	80	61	379.2	267.9	67.0

Coverage calculated assuming a genome size of 12.1Mb.

a Read pool after reads failing quality criteria were trimmed, corrected, or discarded. The relevant procedure is described in *Materials and Methods*.

We aggressively trimmed, corrected, and discarded lower quality reads and assembled those satisfying our quality control criteria using SOAPdenovo (Li *et al.* 2010) and a custom parameter optimization strategy (see *Materials and Methods*). The resulting assemblies exhibited comparable total base counts (Table 2), suggesting that each had converged on the similar physical genome sizes that were predicted by karyotyping and other studies (Fischer *et al.* 2000; Naumov *et al.* 2000; Gonzalez *et al.* 2008). Indeed, the assemblies’ size range of 11.6–11.9 Mb was close to the completed *S. cerevisiae* genome size (12.1 Mb) and bracketed that of the ostensibly completed *S. paradoxus* (Liti *et al.* 2009) (11.7 Mb) assembly, suggesting that our assemblies were essentially complete.

**Table 2:** Genome assembly summary statistics, before and after manual ordering of scaffolds

	Unordered Assembly (Scaffolds)				Ordered Assembly (Ultra-scaffolds)				Percentage of Assembly*a*
	Number	Bases	N50	Gaps	Number	Bases	N50	Gaps	
*S. bayanus* (CBS 7001)	629 (147)	11,668,028	444,551	380	16	11,467,582	905,555	394	98.3%
*S. kudriavzevii* (IFO 1802^T^)	1455 (226)	11,736,856	151,185	17	16	11,294,830	882,337	111	96.2%
*S. kudriavzevii* (ZP 591)	1523 (164)	11,642,553	100,201	10	16	11,185,947	882,203	162	96.2%
*S. mikatae* (IFO 1815^T^)	1220 (159)	11,922,798	360,232	18	16	11,445,471	800,823	52	96.0%

Numbers in parentheses indicate scaffolds longer than 500 bp.

a Percentage of base pairs in the unordered assembly that are also present in the ordered assembly. Neither contigs with an average Kmer coverage less than 20 nor gaps in scaffolds (*i.e.*, N bases) were counted toward assembly statistics.

The new assemblies also had many fewer gaps and greatly improved continuity compared to the originally published versions. For instance, our unordered *S. mikatae* assembly consisted of 1220 scaffolds, of which 159 were longer than 500 bp, and many of the shorter ones were likely to be spurious byproducts of the short-read assembly process. Notably, those 159 scaffolds accounted for >98% of the assembly and contained just 18 gaps (Table 2). By contrast, the previous assemblies consisted of more than 300 scaffolds longer than 500 bp, and these contained >1300 gaps averaging >600 bp. Indeed, the contig N50s were on the order of 20 kb compared to our scaffold N50 of >360 kb (a fair comparison given the paucity of gaps in our assembly). Though the relative improvement varied by species, the N50 range (151–445 kb) of our unordered assemblies represented significant improvements in all cases (Table 2).

Excluding sub-telomeric regions, five *S. bayanus* chromosomes (I, VI, XI, XII, XIII) were each represented by a single large scaffold. The same was true for five *S. mikatae* chromosomes (II, VII, VIII, XI, XVI), and because of the paucity of gaps in this assembly the sequences were almost completely contiguous (note that in all species other than *S. cerevisiae* a gap persists at the rDNA locus on ChrXII). These observations, and the small number of rearrangements believed to distinguish the karyotypes of *Saccharomyces sensu stricto* yeasts (Fischer *et al.* 2000), prompted us to use sequence similarity to organize our initial assemblies with respect to the *S. cerevisiae* genome. By ordering and orienting 46–154 scaffolds per assembly from MEGABLAST results, we were able to organize 96–98% of bases in each species into 16 ultra-scaffolds, which were likely collinear with chromosomes (Table 2). As *S. bayanus* and *S. mikatae* have translocations relative to *S. cerevisiae*, we numbered all ultra-scaffolds/chromosomes according to which centromere they contained (defined largely by flanking synteny with *S. cerevisiae*; supporting information, Figure S1). This nomenclature differs from that proposed by Fischer *et al.*, but is simpler and hopefully acceptable to the community.

In creating ultra-scaffolds for each species, we had little difficulty observing the two known translocations in *S. mikatae* IFO 1815^T^ and four translocations in *S. bayanus* CBS 7001 (Fischer *et al.* 2000). Indeed, most translocation breakpoints were spanned by large scaffolds, and were immediately visible in the scaffold alignments to the *S. cerevisiae* genome. As previously observed (Fischer *et al.* 2000), the genomes of both *S. kudriavzevii* strains appeared completely collinear with the *S. cerevisiae* genome. These observations suggested that the new assemblies were free of gross assembly errors. To test whether our proposed ultra-scaffolds had erroneously linked genomic segments, we designed PCR primers spanning 32 proposed junctions in *S. bayanus*. Twenty-nine of these primer sets (91%) yielded PCR products of appropriate sizes (0.5–4 kb), and the remainder likely failed due to the length of the intervening gap (data not shown).

Despite the overall improvements in the genome assemblies, several systematic differences were evident. Notably, the unordered *S. kudriavzevii* and *S. mikatae* assemblies contained many fewer gaps than the *S. bayanus* assembly whereas the unordered *S. kudriavzevii* assemblies had considerably smaller N50 values (though still an order of magnitude greater than the original 2003 assemblies). These results were direct consequences of the mixture of read types used to assemble each strain. For instance, the greater N50 obtained for *S. bayanus* and *S. mikatae* than for *S. kudriavzevii* IFO 1802^T^ was due to the availability of two long-insert libraries in the first two cases (∼90,000 read pairs) but only one library in the latter (∼27,000 read pairs). On the other hand, the higher number of gaps in the unordered *S. bayanus* assembly relative to *S. mikatae* and *S. kudriavzevii* IFO 1802^T^ is likely due to the lower Kmer size used to assemble the Illumina reads (31 bp), and the somewhat lower Kmer coverage (45.1×; Table 1). [Kmer is the length of DNA that is used to construct the de Bruijn graph during assembly, and is the minimum number of identical bases required to join two overlapping reads (Zerbino and Birney 2008).] By contrast, in the case of both *S. mikatae* and *S. kudriavzevii* our raw short-read coverage of 262.1–362.3× translated to 67.0–98.4× Kmer coverage at a Kmer size of 61 bp. Given the close tracking between Kmer size and gap number, we believe that many of the remaining gaps in the *S. bayanus* assembly were likely to be between 31 and 61 bp in length and thus to represent minimal obstacles to routine use. Indeed, in annotating the new *S. bayanus* assembly it was clear that most gaps caused small interruptions in gene sequences rather than gene absences.

### Telomeres, transposons, and other repeats: an assembly challenge

Telomeres, transposons, and other long and highly repetitive sequences such as mammalian centromeres remain one of the main stumbling blocks to the assembly of truly complete genomes. To assemble and place any given repetitive sequence correctly, one must have library inserts of a length greater than the length of that sequence as well as unique DNA sequence on at least one side. Our Illumina libraries had mean insert sizes of 200–440 bp, which is considerably shorter than full-length transposons in *S. cerevisiae* (*e.g.*, full-length Ty1 elements are ∼6 kb long). In *S. cerevisiae*, sub-telomeric duplication blocks and repeats that possess few sequence differences can reach 30kb in size (Louis 1995). The combination of the high-coverage Illumina reads with the lower-coverage Sanger shotgun reads (4–5 kb insert sizes) allowed us to assemble many subtelomeric sequences, but they remained much more fragmented than the rest of the genome. Indeed, although all of our assemblies extended into the subtelomeric regions of most chromosomes, few scaffolds appeared to reach the telomeric terminal repeats. The two best examples, Skud_66 (IFO 1802^T^) and Skud_52 (ZP 591), were large *S. kudriavzevii* scaffolds (>50 kb) that corresponded along most of their lengths to *S. cerevisiae* ChrIV (right sub-telomere) and ChrVII (left sub-telomere), respectively. However, near the telomere end of each scaffold, the synteny with *S. cerevisiae* chromosomes is broken, leaving multiple apparent rearrangements with other telomeres. In general, synteny was poorly conserved in the sub-telomeric regions of all three species.

The ability to assemble the telomeric regions correlated with Illumina read length, even when the Kmer length used for two of the assemblies was identical. *S. kudriavzevii*, with a read length of 114 bases and Kmer size of 61, had nine scaffolds with terminal-repeat sequences, including the two completely assembled telomeres described above. *S. mikatae*, with a read length of 80 bases and Kmer size of 61, had only one small scaffold containing terminal-repeat sequences. *S. bayanus*, with a read length of 51 bases and Kmer size of 31, had no terminal-repeat sequences in its genome assembly. These data suggest that read length and library insert size were both limiting factors to assembling repetitive regions with current genome assembly software. All of our raw reads are publicly available (http://ncbi.nlm.nih.gov/sra; http://www.SaccharomycesSensuStricto.org), and we urge interested members of the genomics community to use them to develop methods to improve the genome assemblies of these and other problematic regions.

### Updated genome annotations and identification of syntenic orthologs

To provide a basis for future comparative functional studies, we annotated tRNAs, repeats, centromeres, and protein-coding genes in the new genome assemblies. We anticipate the addition of more classes of functional elements in the future. For the present annotation, we took two steps to generate a robust set of protein-coding gene predictions. First, we used a yeast splice-site model as well as homology-assisted gene prediction to ensure correctly delimited genes with introns or with reading-frame interruptions. This step allowed us to correctly recover genes that have traditionally not been well annotated. For example, *RPS7B* (*YHR021C*) and *BOS1* (*YLR078C*) both have first exons that are just 3 bp long, an intron (550 bp and 87 bp respectively) and a larger second exon. In the case of *RPS7B* the intron is more than twice as long as the reading frame, which is only 249 bp—below the 100 AA (300 bp) length minimum that is a common threshold. In both cases we detected a strong match to our splice site model that allowed us to recover homologous gene structures in all five species. Similarly, we predicted homologous structures consisting of three exons and two introns for *SUS1* (*YBR111W-A*) in all five species.

To distinguish spurious open reading frames from biologically meaningful ones, we developed a database of HMMER3 protein hidden Markov models (HMMs) based on the Yeast Gene Order Browser (YGOB) (Byrne and Wolfe 2005). HMMER3 exhibits significantly increased sensitivity and specificity compared to older tools such as BLAST and combined with the gold standard human-curated YGOB database (Byrne and Wolfe 2005; Wapinski *et al.* 2007a) provides a powerful basis for recognizing small or fast-evolving genes and for distinguishing closely related ones. Using this approach, we could detect small genes that can easily go undetected such as the *MFA1* gene (*YDR461W*, which encodes a 37AA-long mating pheromone **a**-factor protein) and the *PMP2* gene (*YEL017C-A*, which encodes a 40AA protein). We also detected rapidly evolving genes such as *SIR4* (*YDR227W*) (Zill *et al.* 2010) and *YSW1* (*YBR148W*) (Kellis *et al.* 2003) with high confidence in all species. Altogether, we detected between 5440 and 5559 genes with homology to one of our YGOB-HMMs (Table 3, Table S2) and no more than 100 of the 4704 families represented by a YGOB-HMM were absent from any genome (the one exception was *S. paradoxus*, which had some remaining large gaps). Thus, these genes defined the core yeast proteome.

**Table 3:** Counts of annotated tRNA and protein-coding genes across representative strains of five Saccharomyces species

	tRNAs	Protein-Coding Genes (by Homology)*a*			Total
		YGOB	SGD	Other	
*S. cerevisiae*	275	5490	881	33	6679
*S. paradoxus*	273	5440	745	46	6504
*S. mikatae*	291	5454	510	51	6306
*S. kudriavzevii*	280	5450	409	48	6187
*S. bayanus*	279	5559	432	48	6318
Orthogroups*^b^*	229	5141	120	0	5490

*S. kudriavzevii* is represented by IFO 1802^T^.

^a^ Protein-coding gene counts are subdivided by homology to families in the Yeast Gene Order Browser (YGOB) (Byrne and Wolfe 2005), genes annotated in the *Saccharomyces* Genome Database (SGD) (Engel *et al.* 2010), or other protein databases (Other) (see *Materials and Methods*).

^b^ Each column shows the number of genes for which syntenic orthologs were detected in all five species.

We also detected several hundred genes in each genome with sequence similarity to a gene in the *Saccharomyces* Genome Database (SGD, http://www.yeastgenome.org) but not to a YGOB-HMM (Table 3). In contrast to the consistent number of genes with homology to YGOB-HMMs, the recovery of genes with homology to an SGD-only gene declined rapidly with evolutionary distance from *S. cerevisiae* (Table 3). Although some of these are located in subtelomeres, many are annotated as dubious by SGD suggesting they are not biologically relevant (Kellis *et al.* 2003). The best sequence-based method to determine whether a gene is real is to test if dN/dS (ω) is significantly different from 1; however, we were able to recover complete ortholog sets for only a small fraction of these genes (Table 3) and hence this test was not performed. Finally, we also predicted a small number of open reading frames that we could associate neither to YGOB-HMMs nor to a known SGD gene but for which we found some evidence of function (see *Materials and Methods*). On closer inspection, the majority of these were related either to transposable elements or derived from Y´ elements, but some appeared to be species-specific genes (discussed further below). Taken together, our reannotation suggested that *Saccharomyces sensu stricto* yeasts share a large common proteome with other yeast species (Dujon *et al.* 2004; Souciet *et al.* 2009) but also possess a much smaller set of genes that distinguish them from other yeasts and from each other.

To facilitate future comparative studies, we used our revised gene annotation to identify sets of genes that are orthologous across the representative strains of all five species. By making extensive use of homology and synteny (see *Materials and Methods*), we identified 5261 sets of orthologous proteins (82–87% of predicted protein-coding genes; Table S1) as well as 229 tRNAs at syntenic locations, for a total of 5487 complete orthologous gene sets (Table 3). (We note that 5141 of the 5261 protein-coding orthogroups had YGOB support.) This analysis approximately doubled the number of five-species syntenic orthologs that were previously available (2742) (Cliften *et al.* 2003; Kellis *et al.* 2003), and also dramatically expanded the previous Kellis *et al.* dataset that identified 4180 orthologs but did not include *S. kudriavzevii*. The nearly complete genomes and improved annotations can be visually perused on a Gbrowse site (available at http://www.SaccharomycesSensuStricto.org).

### Evolutionary analyses of a nearly complete set of Saccharomyces orthologs

Our expanded ortholog dataset (5261 orthogroups) allowed examination of the selection pressures operating on yeast protein-coding genes on a genome-scale, painting a broad-brushstroke picture of how yeast genes evolve. This portrait should be considered conservative because of the absence of some subtelomeric genes from our ortholog set, and because of the filters we applied, which removed orthologs with problematic alignments (see *Materials and Methods*). We calculated the average ω ratio of nonsynonymous (dN) to synonymous (dS) substitutions (dN/dS ratio) for each of 5152 unique (1:1:1:1:1) orthologs conserved across all five species. No high-quality ortholog achieved an average ω value of 1, with the fastest evolving gene (*YBR184W*) having ω = 0.58 (File S1). The average across all high-quality orthologs was ω = 0.10, consistent with previous studies (Kellis *et al.* 2003), and suggesting that most yeast genes are subject to strong purifying selection.

To examine yeast gene evolution on a finer scale, we considered variation in selection pressure across the *Saccharomyces sensu stricto* genus by comparing three alternative hypotheses of the distribution of the ω ratio (dN/dS) along the phylogeny (Figure 2A, File S1). Specifically, for each set of unique orthologs conserved across all five species, we evaluated the hypothesis that all branches of the phylogeny exhibited the same ω ratio (H_0_) against a set of alternative hypotheses (H_1_) under which the ω ratio along the external branch for a given species was different from that in the rest of the branches of the phylogeny (H_1_ hypotheses; Figure 2A). To discriminate between genes that exhibited a different ω ratio only along a branch leading to a particular species and genes that had distinct ω ratios for all branches of the phylogeny, we also tested the set of H_1_ hypotheses against the hypothesis in which each lineage exhibited its own ω ratio (H_2_; Figure 2A). As expected, the overwhelming majority of genes did not reject the null hypothesis of a uniform ω ratio across the phylogeny, but 107–251 genes in each species exhibited statistically significant lineage-specific ω ratios (Figure 2B). For example, our results indicated that 152 *S. cerevisiae* genes showed lineage-specific ω ratios. Of those 152 genes, 65 supported a higher ω ratio in the *S. cerevisiae* branch relative to the rest of the phylogeny, whereas 87 genes supported a lower ω ratio. The complete list of candidate genes that exhibit lineage-specific ω ratios is provided in File S1.

We also examined each of the 5152 orthologs for evidence of positive selection by comparing two alternative models (called M7 and M8) of the distribution of ω that differ with respect to the allowance of a subset of codon sites to be under positive selection (with ω > 1). We found 123 genes (after filtering, see *Materials and Methods*) whose sequence evolution fit a model of codon evolution in which a detectable fraction of sites has been under positive selection (M8) better than it fit a model where sites evolve neutrally (M7) (File S1). Rapidly evolving genes are more likely to have functionally diverged, potentially contributing to genetic incompatibilities between species (Orr 2009). Among the 123 genes found in our analysis was *SIR4*, which has previously been shown to be under positive selection by multiple measures (Zill *et al.* 2010; Zill *et al.*, unpublished data), supporting the view that codons within these 123 genes were evolving faster than neutral, and were not simply misaligned. Several genes involved in mitochondrial maintenance and inheritance (*e.g.*, *QRI7*, and *AIM2*, *AIM14*, *AIM21*, *AIM43*) fit this pattern, consistent with suggestions that divergence in nuclear genes with mitochondrial functions have contributed to speciation in *Saccharomyces* (via cytonuclear incompatibility) (Chou and Leu 2010; Lee *et al.* 2008). Intriguingly, we also found several meiotic genes involved in homologous chromosome interactions, and/or DNA-repair-coupled chromatin modifications during meiosis, that were undergoing positive selection (*e.g.*, *ZIP2*, *PDS5*, *SRS2*, *DOT1*, and *ESC2*). *Saccharomyces sensu stricto* species are post-zygotically isolated due to a failure of inter-species homologous chromosomes to segregate properly in meiosis I, which is caused in part by nucleotide sequence divergence acted on by the mismatch repair machinery (Greig *et al.* 2003; Liti *et al.* 2006). It is possible that these rapidly diverging chromosome-biology genes play a role in the meiotic barrier between species.

### A relative timescale of interspecies divergence

**Figure 3:**
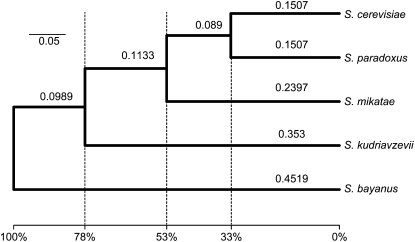
Relaxed molecular clock estimation of relative species divergence within the *Saccharomyces sensu stricto* genus. The top scale bar and the values above branches denote estimated substitutions per site. The bottom scale bar expresses species divergence in percentage points relative to the origin of the genus.

To establish a quantitative framework for interspecies divergence in the *Saccharomyces sensu stricto* genus, we used a relaxed molecular clock approach to estimate the relative divergence times among lineages (Drummond *et al.* 2006). However, because we were unable to consistently estimate the necessary parameters with our complete set of orthogroups (data not shown), we analyzed a smaller dataset of 106 genes spanning the yeast genome (Rokas *et al.* 2003). As the origin of the genus coincides with the divergence of *S. bayanus* from the rest of the *Saccharomyces sensu stricto* lineage, our results showed that the divergence of *S. kudriavzevii* was 78% as old as the lineage and the divergence of *S. mikatae* was 53% as old, whereas the divergence of *S. cerevisiae* and *S. paradoxus* was 33% as old as the lineage (Figure 3).

### Species-specific gene gains and losses

The improved assemblies and consequent improved ability to detect orthologs in all five species allowed us to revisit which genes had been gained and lost in specific lineages (see *Materials and Methods*). Below we present the results of our initial five-genome survey as a set of hypotheses, in which each candidate genetic difference (such as a species-specific gene loss) predicts an alteration of the ancestral *Saccharomyces* genetic network. To organize lineage-specific gene-level changes into a simple, logical framework, we first considered any genes that were present in the common ancestor of these yeasts but were not found in all five of the modern genomes as “losses.” (We note that in some cases we were able to detect pseudogenes or truncated genes but in other cases the genes had essentially vanished, consistent with either large deletion events or the accumulation of many smaller changes.) We next divided the losses into three sub-categories: “lineage-restricted losses” (lost in one or two species), “widespread losses” (absent from more than two species, but may have involved more than one loss event), and losses of one paralog of a duplicate gene pair descended from the whole-genome duplication (“duplicate gene losses”). Any genes present in one or more of the five *Saccharomyces* species but not in the *sensu stricto* ancestor we considered to be “gains.” We also analyzed tRNA variation in each of the five species.

#### Lineage-restricted losses:

We identified at least 44 examples of genes lost from only one or two lineages (File S2). One interesting example of a gene that appeared to be completely missing was the loss from *S. cerevisiae* of a GATA family transcription-factor gene [Anc_2.395; we use the nomenclature proposed by YGOB to identify conserved yeast loci to also refer to the derived YGOB-HMM (Gordon *et al.* 2009)] related to *GAT3* and *GAT4*. This loss suggested that a suite of target genes may have experienced regulatory changes relative to the *Saccharomyces* ancestral circuit. In another example, *S. mikatae* has lost *PDC6*, which encodes a minor pyruvate decarboxylase expressed under sulfur limitation. This gene has additionally been pseudogenized in *S. kudriavzevii*, suggesting that these species may have experienced selective pressure to alter their alcohol metabolism.

In addition to identifying genes that were entirely missing, we detected many pseudogenes with varying numbers of reading-frame disruptions. A well-known example of this sort of mutation is the parallel inactivation (as pseudogenes) of all the *GAL* genes in *S. kudriavzevii* IFO 1802^T^ but not ZP 591 (Hittinger *et al.* 2004; Hittinger *et al.* 2010). Our analysis recovered all of these and a previously described mutation in the *S. bayanus* CBS 7001 *BAR1* gene (Zill and Rine 2008), where a single base-pair deletion leads to a frameshift and truncated coding sequence. We found several additional likely pseudogenes in each of the five species (File S2). Notable among these were several metabolic genes such as *GTO1*, which encodes an omega-class glutathione transferase, lost in *S. bayanus* and *S. mikatae*. Similarly, *S. mikatae* lost the genes *OYE3*, which encodes an NADPH oxidoreductase, and *GND2*, which encodes the minor isoform of 6-phosphogluconate dehydrogenase. Additionally, a few losses involve genes important for stress responses or environmental interactions such as *S. bayanus CAD1*, which encodes an AP-1-like basic leucine zipper (bZIP) transcription factor involved in stress responses and iron metabolism.

#### Widespread losses:

Many differences among species that initially appeared to be lineage-specific gains were in fact losses of ancestral genes in multiple lineages. For example, it was clear from the presence of a syntenic homolog in *Naumovozyma castellii* (syn. *Saccharomyces castellii*) and other yeast species that the budding-yeast *Dicer* homolog in *S. bayanus* (Anc_8.880) (Drinnenberg *et al.* 2009) has been lost in the other *sensu stricto* species, and not gained on the *S. bayanus* lineage. The same conclusion applied to several genes with sequence similarity to a YGOB-HMM but for which we had no functional data (Table 4). The rapidly evolving gene *Sbay_15.267* also fell into this category; orthologs were found in *Candida glabrata* (*Nakaseomyces* clade) *N. castellii*, and *S. bayanus* but not other sequenced species. The average dN/dS value was estimated as 0.33, but only about half of the codons could be aligned in all three species, indicating that despite the conservation of an intact open reading frame it was likely one of the fastest-evolving genes in yeast (Zill *et al.* 2010). We also detected the multiple paralogs of *SIR1* in *S. kudriavzevii* and *S. bayanus*, which function in transcriptional silencing in these species (Gallagher *et al.* 2009).

**Table 4:** Genes not previously reported in the *Saccharomyces sensu stricto*

*Representative* Gene(s)	Homolog	Presence Pattern*a*	Functional Annotation
*Smik_18.9*	KLTH0F00110	0:0:1:0:0	*S*-adenosylmethionine-dependent methyltransferase; weak homolgy to Anc_8.241
*Sbay_15.364*	Anc_5.74	0:0:0:0:1	Uncharacterized
(*YJR107C-A*)	Anc_7.495	1:1:1:1:1	Not annotated in SGD. dN/dS = 0.29; between *YJR107W/YJR108W*
*Sbay_10.240*	Anc_8.350	0:0:0:0:1	Uncharacterized
*Spar_6.12*	Anc_8.663	0:1:1:1:1	Nonsyntenic; uncharacterized
*Sbay_13.12*	Anc_8.869	0:0:0:0:1	Uncharacterized
*Sbay_13.48*	Anc_8.880	0:0:0:0:1	Endoribonuclease in the RNase III family (budding yeast *Dicer*)*b*
*Sbay_15.267*	CAGL0J10714g	0:0:0:0:1	Syntenic homolog. dN/dS = 0.33; also annotated in *N. castellii*
*Smik_10.15*	RCFBP_mp20323	0:0:1:0:0	NTF2_like superfamily; similar to RCFBP_mp20323 from *Ralstonia solanacearum*
*Smik_29.1/Spar_12.256*	CGSSp3BS71_00010	0:1:1:0:0	Similar to CGSSp3BS71_00010 from *Streptococcus pneumoniae*
*Sbay_15.427*	Kwal_8.576	0:0:0:0:1	Nitrilase superfamily
*Sbay_17.1*	SAKL0C00330g	0:0:0:0:1	Hyphal_reg_CWP superfamily

^a^ Number of detected copies in *S. cerevisiae*, *S. paradoxus*, *S. mikatae*, *S. kudriavzevii* (IFO1802^T^), and *S. bayanus*, respectively.

^b^ Budding yeast *Dicer* was described in (Drinnenberg *et al.* 2009)

#### Duplicate gene losses:

As a whole-genome duplication (WGD) event occurred in the ancestry of the *Saccharomyces sensu stricto* yeasts, we paid special attention to detecting duplicate pairs originating from this event. In total, our automated procedure detected 1044–1084 WGD duplicate genes (522–542 pairs) in each species (see *Materials and Methods*). 98% of those predicted in *S. cerevisiae* agreed with those listed in the Yeast Gene Order Browser (Byrne and Wolfe 2005). From these data, it was apparent that many of the potential gains and losses we detected were actually the result of differential loss of ancestrally duplicated genes from the yeast whole-genome duplication (Table 5), a process that contributes to reproductive isolation (Lynch and Conery 2000; Scannell *et al.* 2006). For example, it was clear from the syntenic context that the presence of two copies of *GAL80* in *S. bayanus* was not due to a recent duplication but to loss of one of the two ancestral copies derived from the WGD on the shared *S. cerevisiae/S. kudriavzevii* lineage and its retention on the *S. bayanus* lineage (Hittinger *et al.* 2004; Cliften *et al.* 2006). Indeed, both *GAL80* WGD duplicates were also retained in other yeasts such as *N. castellii*. Interestingly, not all of the cases in Table 5 can be explained by a single loss event. For example, the gene *YML020W* was present in two copies in *S. paradoxus*, indicating that it was present in duplicate until relatively recently. Therefore, one copy has become a pseudogene independently in each of the other *Saccharomyces sensu stricto* lineages. The same logic applied for *SSU1*. These observations were in line with previous predictions (Scannell *et al.* 2007b).

**Table 5:** Loss of duplicate genes from the ancient whole-genome duplication in the *Saccharomyces sensu stricto* clade

*S. cerevisiae* Gene(s)	YGOB Locus	Retention Pattern*a*	Functional Annotation
*YCL048W-A / YDR524C-B*	Anc_1.22	2:1:1:1:2	Uncharacterized
*YFR017C / YOL024W*	Anc_1.363	2:2:1:2:2	Predicted to have thiol-disulfide oxidoreductase active site
*ECM10/SSC1*	Anc_1.474	2:2:1:2:2	Hsp70 family; localized in mitochondrial nucleoids; plays a role in protein translocation
*GAL80*	Anc_1.500	1:1:1:1:2	Inhibits transcriptional activation by Gal4p
*HEK2*	Anc_3.318	1:1:1/ψ:2:2	RNA binding protein with similarity to hnRNP-K; localizes to the cytoplasm and subtelomeric DNA
*PMT4*	Anc_4.379	1:1:1:1:2	Protein amino acid O-linked glycosylation
*SLT2 / YKL161C*	Anc_5.274	2:2:1:2:2	Serine/threonine MAP kinase involved in regulating the maintenance of cell wall integrity
*CAD1/YAP1*	Anc_5.528	2:2:2:2:1	AP-1-like basic leucine zipper (bZIP) transcriptional activator involved in stress responses, iron metabolism, and pleiotropic drug resistance
*YML020W*	Anc_5.554	1:2:1/ψ:1/ψ:1/ψ	Uncharacterized
*YDR066C / YER139C*	Anc_8.181	2:2:2:1:2	Uncharacterized
*SSU1*	Anc_8.569	1:2:1:1:1	Plasma membrane sulfite pump
*ARL1*	Anc_8.597	1/ψ:1/ψ:1:1/ψ:2	Soluble GTPase with a role in regulation of membrane traffic
*URA5/URA10*	Anc_8.827	2:2:1:2:2	Phosphoribosyltransferase; fifth step in pyrimidine biosynthesis pathway

^a^ Number of detected copies or pseudogenes (ψ) in *S. cerevisiae*, *S. paradoxus*, *S. mikatae*, *S. kudriavzevii*, and *S. bayanus*, respectively.

#### Gene gains:

Although gene gains are quite rare in hemiascomycetes (Hall and Dietrich 2007; Gordon *et al.* 2009), we found three candidates for horizontal gene transfer events. For example, *Smik_18.9* is 849 bp long and has codon usage typical of other *S. mikatae* genes but has no detectable homology to any gene in SGD. On closer inspection, it shows weak sequence similarity to the YGOB-HMM Anc_8.241 (homologous to *CRG1*/*YHR209W* and *TMT1*/*YER175C*) and is clearly related to the *Lachancea thermotolerans* gene, KLTH0F00110. Both KLTH0F00110 and *CRG1* are annotated as *S*-adenosylmethionine-dependent methyltransferases, indicating that *Smik_18.9* is a real gene that likely contributes to species-specific biology. Of the two other possible horizontal gene transfers in *S. mikatae*, one, CGSSp3BS71_00010 (an uncharacterized protein from *Streptococcus pneumoniae*), was apparently also present in *S. paradoxus*. These bacterial sequences were not merely contaminants introduced during Illumina library preparation, as we found identical sequences using BLAST in the Sanger shotgun reads. However, as there was only a single hit in NCBI, we could not construct gene trees to test whether it was a true horizontal transfer. Similarly, the *S. mikatae* gene *Smik_10.15* was a candidate horizontal gene transfer, because it displayed strong sequence similarity to a gene with an NTF2 domain from the bacterium *Ralstonia solanacearum* (E-value of 6E-18), but no fungal homolog was detected. However, because *Smik_10.15* could not be placed within a specific bacterial clade (data not shown), we have not yet confirmed this as a horizontal transfer event.

Interestingly, our analysis led to the discovery of a novel *S. cerevisiae* gene homologous to Anc_7.495. This gene is conserved across all *Saccharomyces sensu stricto* (as well as more diverged) yeasts but was previously not observed, presumably due to its short length (237 bp; Table 4). A dN/dS ratio of 0.29 confirms that this gene is subject to codon-level evolutionary constraint within the *Saccharomyces sensu stricto* and, based on the established naming convention, we propose that it be named *YJR107C-A*.

#### tRNA Variation:

Using tRNA-ScanSE, we identified all 275 SGD-annotated tRNA genes in the *S. cerevisiae* genome with no false positives. Given the reliability of this procedure, we were surprised to observe that the number of Ser:AGA tRNA genes varied from 8 in *S. kudriavzevii* to 14 in *S. bayanus* (Table S3). Based on synteny, we estimate that there were 9–10 copies in the ancestor of all the species presented here and that whereas *S. kudriavzevii* has sustained a net loss of Ser:AGA tRNA genes, *S. bayanus* and *S. paradoxus* have gained copies. Variation in Ser:AGA tRNA copy number was not compensated by variation in the copy number of other serine tRNA genes (Table S3).

### Genetically tractable strains for *S. mikatae*, *S. kudriavzevii*, and *S. bayanus*

Functional tests of the genetic rewiring and other hypotheses presented above would require genetically tractable and marked strains from across the *Saccharomyces* genus. The four strains whose genomes we sequenced (IFO 1815^T^ and derivatives of IFO 1802^T^, ZP 591, and CBS 7001) were originally prototrophic and homothallic. To enable genetic experiments to be conducted with similar ease to experiments in *S. cerevisiae*, the *HO* gene was inactivated and auxotrophic markers were introduced into the reference or type strains for *S. mikatae*, *S. kudriavzevii*, and *S. bayanus*. The *ura3∆0* strains are of particular utility because they enable the two-step procedure necessary to introduce precise changes in individual nucleotides (Storici *et al.* 2001). Although some of these strains have been described previously (Hittinger *et al.* 2010; Zill *et al.* 2010; Gallagher *et al.* 2009), for the community’s benefit, we briefly summarize below how each set of strains was generated (see *Materials and Methods*). A convenient collection of the most useful strains can be obtained from a single central repository (Table 6). Some heterothallic, marked *S. paradoxus* strains are already available (Cubillos *et al.* 2009). Collectively, laboratory-ready strains are now available for genetic experiments in every *Saccharomyces* species whose genome sequence has been published.

**Table 6:** Construction of heterothallic haploid strains with auxotrophic markers for *S. mikatae*, S. *kudriavzevii* and *S. bayanus*

Species	Strain	Original	Genotype	Reference
*S. mikatae*	JRY9171	IFO 1815^T^	*MAT****a*** *hoΔ::KanMX ura3Δ::HygMX*	This study
*S. mikatae*	JRY9172	IFO 1815^T^	*MATα hoΔ::KanMX ura3Δ::HygMX*	This study
*S. mikatae*	JRY9173	IFO 1815^T^	*MAT****a*** *hoΔ::NatMX ura3Δ::HygMX*	This study
*S. mikatae*	JRY9174	IFO 1815^T^	*MATα hoΔ::NatMX ura3Δ::HygMX*	This study
*S. mikatae*	JRY9175	IFO 1815^T^	*MAT****a*** *hoΔ::KanMX his3Δ::HygMX*	This study
*S. mikatae*	JRY9176	IFO 1815^T^	*MATα hoΔ::NatMX trp1Δ::HygMX*	This study
*S. mikatae*	JRY9177	IFO 1815^T^	*MAT****a*** *hoΔ::KanMX his3Δ::HygMX ura3Δ::HygMX*	This study
*S. mikatae*	JRY9178	IFO 1815^T^	*MATα hoΔ::KanMX his3Δ::HygMX ura3Δ::HygMX*	This study
*S. mikatae*	JRY9179	IFO 1815^T^	*MAT****a*** *hoΔ::NatMX his3Δ::HygMX ura3Δ::HygMX*	This study
*S. mikatae*	JRY9180	IFO 1815^T^	*MATα hoΔ::NatMX his3Δ::HygMX ura3Δ::HygMX*	This study
*S. mikatae*	JRY9181	IFO 1815^T^	*MAT****a*** *hoΔ::KanMX trp1Δ::HygMX ura3Δ::HygMX*	This study
*S. mikatae*	JRY9182	IFO 1815^T^	*MATα hoΔ::KanMX trp1Δ::HygMX ura3Δ::HygMX*	This study
*S. mikatae*	JRY9183	IFO 1815^T^	*MAT****a*** *hoΔ::NatMX trp1Δ::HygMX ura3Δ::HygMX*	This study
*S. mikatae*	JRY9184	IFO 1815^T^	*MATα hoΔ::NatMX trp1Δ::HygMX ura3Δ::HygMX*	This study
*S. kudriavzevii*	FM1097	IFO 1802^T^	*MAT*α *hoΔ::natMX*	Hittinger *et al.* 2010
*S. kudriavzevii*	FM1098	IFO 1802^T^	*MAT****a*** *hoΔ::natMX*	Hittinger *et al.* 2010
*S. kudriavzevii*	FM1363	IFO 1802^T^	*MAT*α *hoΔ::kanMX*	This study
*S. kudriavzevii*	FM1403	IFO 1802^T^	*MAT****a****/MAT*α *ho*Δ*::kanMX/hoΔ::kanMX*	This study
*S. kudriavzevii*	FM1122	IFO 1802^T^	*MAT*α *hoΔ::natMX ura3Δ0*	This study
*S. kudriavzevii*	FM1141	IFO 1802^T^	*MAT*α *hoΔ::natMX ura3Δ0 trp1Δ::ScerURA3^+^*	This study
*S. kudriavzevii*	FM1388	IFO 1802^T^	*MAT*α *hoΔ::natMX ura3Δ0 his3Δ0*	This study
*S. kudriavzevii*	JRY9185	IFO 1802^T^	*MAT****a*** *hoΔ::natMX ura3Δ0*	This study
*S. kudriavzevii*	JRY9186	IFO 1802^T^	*MAT*α *hoΔ::natMX trp1Δ0*	This study
*S. kudriavzevii*	JRY9187	IFO 1802^T^	*MAT****a*** *hoΔ::natMX trp1Δ0 ura3Δ0*	This study
*S. kudriavzevii*	JRY9188	IFO 1802^T^	*MAT*α *hoΔ::natMX trp1Δ0 ura3Δ0*	This study
*S. kudriavzevii*	FM1109	ZP 591	*MAT****a*** *hoΔ::kanMX*	Hittinger *et al.* 2010
*S. kudriavzevii*	FM1110	ZP 591	*MAT*α *hoΔ::kanMX*	Hittinger *et al.* 2010
*S. kudriavzevii*	FM1071	ZP 591	*MAT****a****/MAT*α	Hittinger *et al.* 2010
*S. kudriavzevii*	FM1158	ZP 591	*MAT****a****/MAT*α	This study
*S. kudriavzevii*	FM1400	ZP 591	*MAT****a****/MAT*α *ho*Δ*::kanMX/hoΔ::kanMX*	This study
*S. kudriavzevii*	FM1340	ZP 591	*MAT****a*** *hoΔ::natMX ura3Δ0*	Hittinger *et al.* 2010
*S. kudriavzevii*	FM1123	ZP 591	*MAT****a*** *hoΔ::kanMX ura3Δ0*	Hittinger *et al.* 2010
*S. kudriavzevii*	FM1192	ZP 591	*MAT*α *hoΔ::kanMX ura3Δ0*	This study
*S. kudriavzevii*	FM1194	ZP 591	*MAT****a*** *hoΔ::kanMX trp1Δ0*	This study
*S. kudriavzevii*	FM1131	ZP 591	*MAT*α *hoΔ::kanMX trp1Δ0*	Hittinger *et al.* 2010
*S. kudriavzevii*	FM1183	ZP 591	*MAT****a*** *hoΔ::kanMX ura3Δ0 trp1Δ0*	Hittinger *et al.* 2010
*S. kudriavzevii*	FM1193	ZP 591	*MAT*α *hoΔ::kanMX ura3Δ0 trp1Δ0*	This study
*S. kudriavzevii*	FM1389	ZP 591	*MAT****a*** *hoΔ::kanMX ura3Δ0 his3Δ0*	This study
*S. bayanus*	JRY9189	CBS 7001	*MAT****a*** *hoΔ::NatMX*	This study
*S. bayanus*	JRY9190	CBS 7001	*MATα hoΔ::NatMX*	This study
*S. bayanus*	JRY8149	CBS 7001	*MAT****a*** *hoΔ::NatMX his3 lys2 ura3*	Gallagher *et al.* 2009
*S. bayanus*	JRY8150	CBS 7001	*MATα hoΔ::NatMX his3 lys2 ura3*	Gallagher *et al.* 2009
*S. bayanus*	JRY8153	CBS 7001	*MAT****a*** *hoΔ::NatMX his3 lys2 trp ura3*	Gallagher *et al.* 2009
*S. bayanus*	JRY8154	CBS 7001	*MATα hoΔ::NatMX his3 lys2 trp ura3*	Gallagher *et al.* 2009
*S. bayanus*	JRY8147	CBS 7001	*MAT****a*** *hoΔ::NatMX ade2 his3 lys2 ura3*	Gallagher *et al.* 2009
*S. bayanus*	JRY8148	CBS 7001	*MATα hoΔ::NatMX ade2 his3 lys2 ura3*	Gallagher *et al.* 2009
*S. bayanus*	JRY9191	CBS 7001	*MAT****a*** *hoΔ::NatMX his3 ura3*	This study
*S. bayanus*	JRY9040	CBS 7001	*MAT****a*** *hoΔ::NatMX lys2 ura3*	Zill *et al.* 2010
*S. bayanus*	JRY9192	CBS 7001	*MAT****a*** *hoΔ::NatMX ade2 ura3*	This study
*S. bayanus*	JRY9193	CBS 7001	*MATα hoΔ::NatMX ade2 ura3*	This study
*S. bayanus*	JRY9194	CBS 7001	*MAT****a*** *hoΔ::loxP his3 lys2 ura3*	This study
*S. bayanus*	JRY9195	CBS 7001	*MATα hoΔ::loxP his3 lys2 ura3*	This study

All strains are available upon request from C. T. Hittinger.

### Characteristics of the *Saccharomyces sensu stricto* species that distinguished them from *S. cerevisiae* laboratory strains

Nonmodel species can often pose unanticipated challenges when brought into the laboratory. However, they also offer the great benefit of phenotypic diversity. To highlight some potentially useful characteristics of these newly laboratory-adapted yeast species described above and to alert investigators to potential practical problems, we offer several anecdotal observations culled from our collective experiences working with these nonmodel yeasts. We note that these comparisons are made to standard *S. cerevisiae* laboratory strains such as S288C. Hence some of the trait differences described below almost certainly resulted from the selections imposed on *S. cerevisiae* strains during laboratory adaptation, as others have noted (Liu *et al.* 1996; Gaisne *et al.* 1999).

As with most wild strains, *S. paradoxus*, *S. mikatae*, *S. kudriavzevii*, and *S. bayanus* diploids showed very high levels of sporulation (approaching 100%) when placed on standard potassium acetate medium. These *Saccharomyces sensu stricto* strains also sporulated with striking efficiency (25–50%) after about 1 week on YPD plates stored at room temperature. These strains also sporulated on YPD at 4°C over a somewhat longer period of time. The tetrads of each of these species were of a greater size range than those of *S. cerevisiae*, but were smaller on average. As previously noted, these species preferred to grow at 18–23°C, and did not grow well at 30°C (Sampaio and Goncalves 2008). Indeed, they appeared more sensitive to heat shock than *S. cerevisiae*, but tolerated cold and freeze-thaw cycles better than *S. cerevisiae* (Kvitek *et al.* 2008).

In liquid culture, flocculence was readily apparent in *S. paradoxus* and one of the *S. kudriavzevii* strains (IFO 1802^T^ and derivatives). In fact, IFO 1802^T^ was so flocculent that in overnight liquid culture it grew into spherical, 2-3mm pellets. Flocculence was less pronounced in *S. mikatae*, *S. bayanus*, and *S. kudriavzevii* strain ZP 591, with haploids of these strains more closely resembling the mild clumpiness of *S. cerevisiae* vineyard strain RM11 (R. Brem, personal communication). Interestingly, flocculence in *S. paradoxus* and *S. kudriavzevii* IFO 1802^T^ appeared to be regulated by mating type. In both species, *MAT***a**/α diploids were less flocculent than both *MAT***a** and *MAT*α haploids. This regulation was likely due to mating-type control, rather than diploidy *per se*, as haploid *sirΔ* mutants (which express *HML*α and *HMR***a**) of both species showed reduced flocculence (O. Zill, unpublished data). Cells of all four species displayed obvious differences in size and shape between haploids (small and round, often growing in clusters) and diploids (larger and ovoid, with polar budding). Diploids often appeared bulb-shaped, with a rounded apical tip and a flat base defined by the cell’s site of budding from its mother.

All four species propagated *S. cerevisiae CEN*/*ARS* and 2μ plasmids well enough to conduct complementation experiments (Gallagher *et al.* 2009). However, in *S. kudriavzevii* and *S. bayanus CEN*/*ARS* vectors segregated with lower fidelity than in *S. cerevisiae*, which was likely due to divergence in the *CEN* element (Figure S1; C. T. Hittinger and J. Gallagher, unpublished data). Notably, Japanese *S. kudriavzevii* (IFO 1802^T^) lack functional galactose metabolic and regulatory genes, while Portuguese *S. kudriavzevii* (ZP 591) are Gal^+^ (Hittinger *et al.* 2010). Thus, the standard *GAL* induction plasmids would only work in the Portuguese strains, in which there was still a delayed response to galactose (Hittinger *et al.* 2010). *S. kudriavzevii* was originally reported to utilize the fructose-based complex carbohydrate inulin (Naumov *et al.* 2000). However, others and we were unable to replicate this result (C. T. Hittinger and Gregory I. Lang (Princeton University), unpublished data).

## Discussion

### High-quality Saccharomyces genome assemblies for evolutionary analyses

A vibrant community of geneticists, genomicists, and computational biologists has made *S. cerevisiae* into a model species whose genome is arguably the best described, most easily manipulated, and best understood at all functional levels. Here we have provided a set of genetically tractable laboratory strains and vastly improved genome sequences that make *Saccharomyces sensu stricto* a model genus for evolutionary and comparative analyses and experiments.

Deep paired-end Illumina sequencing allowed us to determine nearly complete genome sequences of *S. mikatae*, *S. kudriavzevii*, and *S. bayanus*, and to assign over 96% of base pairs to specific chromosomal locations. The closure of most gaps and the creation of ultra-scaffolds allowed us to provide a user-friendly genome browser (available through http://www.SaccharomycesSensuStricto.org) for each species that will facilitate rapid experimental design, visualization of data, and further analyses. The ultra-scaffolds should be of particular value to genetic mapping studies. Draft genome sequences of *S. arboricolus* and *S. bayanus* var. *bayanus* (G. Liti, E. Louis, and C. Nieduszynski, personal communication; Libkind, Hittinger *et al.*, unpublished data) are also available, completing the catalog of known species-level diversity for the *Saccharomyces* genus (Figure 1A).

Among model organisms, the genome of *S. cerevisiae* is uniquely well described both in terms of its functional elements and the relationships among those elements. The new assemblies and genetic tools presented here permit the same level of knowledge to be attained in its con-generic species. However, they also open the door to understanding how functions and interactions change over time by studying the same (orthologous) genes in multiple species. Such an evolutionary approach is becoming ever more common and has proven powerful even when applied to pathways with a long history of study in *S. cerevisiae* (Hittinger *et al.* 2010; Zill *et al.* 2010; Lee *et al.* 2008). To facilitate such studies, we have annotated 5261 sets of genes that are orthologous among all five species (Table 3, Table S1). Thus, the vast majority of *Saccharomyces sensu stricto* genes are now available for systematic comparative and evolutionary study.

### The susceptibility of comparative genomics to errors and missing data

The annotation of nearly complete genomes for five *Saccharomyces* species approximately doubled the number of orthologous gene sets available when compared with the sequences available in 2003 (previously 2742 orthogroups). This comparison provides an important reminder of the relationship between assembly completeness, annotation accuracy, and the downstream comparative analyses that rely on complete datasets (*e.g.*, phylogenetics). For example, even if 95% of genes were present and correctly annotated in each assembly, we would only expect 77% (0.95^5^) of genes to be present and correctly assigned to sets of orthologous genes in all species (assuming assembly biases are uncorrelated). Indeed, with five species, genome annotations that were 80% complete and accurate would yield full ortholog sets for just 33% of genes. To obtain orthogroups for 90% of genes would require an average per-genome completeness and accuracy of 98%. Thus, relative to single-genome studies, comparative studies are disproportionately sensitive to missing data and to the quality of the underlying annotations. Because this problem scales exponentially with the number of species, it will become drastically more severe as more species are considered. Therefore, designing procedures and analyses that are robust to missing data must be a key component and priority of future large-scale comparative genome sequencing projects.

### Ancient whole genome duplication still impacts modern yeast evolution

Although we identified orthologs across all five species for most genes, we came across many examples of genes that had been lost in one or more lineages. For example, the losses of *PDC6* on the *S. mikatae* lineage and *CAD1* on the *S. bayanus* lineage immediately suggest potential species-specific biology. Interestingly, many of these losses appeared to involve members of duplicate pairs derived from the yeast whole-genome duplication (Wolfe and Shields 1997). The period after the WGD was characterized by rapid protein evolution (Kellis *et al.* 2004; Scannell and Wolfe 2008) and gene loss (Scannell *et al.* 2006), but our data suggest that duplicate genes continue to experience an elevated rate of loss even 100 million years after the WGD event. Though initially surprising, this conclusion is in line with previous analyses predicting that a small fraction of redundant duplicates remained to be resolved (Scannell *et al.* 2007b). Further, our observations of multiple orthologous losses (*e.g.*, Anc_5.554, Table 5) are consistent with the proposal that if one copy is capable of supplying all the required functions, then the second (“minor”) copy will be convergently lost in all lineages (Scannell and Wolfe 2008). This idea is also supported by the loss of *URA10* rather than *URA5* from *S. mikatae*. *URA5* and *URA10* encode phosphoribosyltransferase genes that catalyze the fifth step in the pathway of *de novo* synthesis of pyrimidine ribonucleotides, but in *S. cerevisiae URA10* supplies less than 20% of the activity and is conditionally expressed. Taken together, these observations point to the fascinating conclusion that the consequences of the WGD are still felt by modern yeast and contribute significantly to genomic and potentially phenotypic differences among *S. cerevisiae* and its con-generic species. Moreover, a specific line of research that emerges from this observation is to compare the biological functions of WGD duplicates that differ in copy number among these five yeasts using the strains that we have generated.

In addition to lineage-specific losses, we also identified a number of candidates for lineage-specific gains, including two possible horizontal gene transfers from bacteria. These genes differentiate the *Saccharomyces sensu stricto* yeasts from one another (Table 4) and thus may play important roles in ecological specialization. Interestingly, among the putative gene gains we identified, was the discovery of a novel gene in *S. cerevisiae* that resides between *YJR107W* and *YJR108W* (Table 4). The biological functions of this rapidly evolving gene are unknown but its deep conservation outside the *Saccharomyces sensu stricto* clade leaves little doubt that it has a function. The discovery of a novel gene in the well-studied *S. cerevisiae* genome validated our goal of producing high-quality annotations for five *sensu stricto* yeasts.

## Conclusions

Along with creating stably marked haploid strains, we have ported routine techniques for manipulating *S. cerevisiae* genetically to the other *Saccharomyces* species. These include the powerful tools of targeted-gene knockouts, plasmid-driven expression, and altering single nucleotides within genomes. Species within the *Saccharomyces sensu stricto* genus also readily hybridize (Masneuf *et al.* 1998; Greig 2009; Martin *et al.* 2009). The complementary markers and mating types in the collection make these experiments especially convenient. Interspecies hybrids can be used in complementation tests to identify mutated genes in species closely related to a model organism (Zill *et al.*, in preparation), and to study the evolution of genetic regulatory circuits (Bullard *et al.* 2010; Gasch *et al.* 2004; Guan *et al.* 2007; Tirosh *et al.* 2009). This expansion of genetically tractable species opens the *Saccharomyces sensu stricto* genus to comparative analysis, and provides molecular biologists with an unrivaled set of tools to explore this model genus.

With near-complete genome sequences, geneticists now have essentially complete experimental access to the genomes of each species. The candidate changes in gene content and selection pressures we found within each *Saccharomyces sensu stricto* species present excellent opportunities to study species-specific biology, and to use these genetic differences to learn how genetic networks have been rewired during the evolution of this genus. At the same time, the large set of orthologs we have identified provides a strong foundation for comparative genetic studies, and should lead to a wealth of discoveries that are refractory to sequence-based analyses. Together, these new genomic resources and universal genetic techniques provide an infrastructure for an unprecedented integration of evolutionary and experimental biology, enabled by the Saccharomyces model genus and the awesome power of yeast genetics.

## Supplementary Material

Supporting Information
