# Supplementary material for: The Awesome Power of Yeast Evolutionary Genetics: New Genome Sequences and Strain Resources for the Saccharomyces sensu stricto Genus
Source: G3 (Bethesda). 2011 Jun 1;1(1):11–25. doi: 10.1534/g3.111.000273 (PMC3276118; doi:10.1534/g3.111.000273)
Supplement: Supporting Information [file supp_1_1_11__index.html]

Supporting Information 

# The Awesome Power of Yeast Evolutionary Genetics: New Genome Sequences and Strain Resources for the *Saccharomyces sensu stricto* Genus

## Supporting Information for Scannell *et al.*, 2011

**Files in this Data Supplement:**

- Supporting Information - Figure S1, Files S1 and S2, and Tables S1-S3 (PDF, 756 KB)
- Figure S1 - Multiple sequence alignment of centromeres from (A) *S. bayanus*, (B) *S. mikatae*, and *S. kudriavzevii* (C) IFO 1802T and (D) ZP 591 (PDF, 588 KB)
- Table S2 - YGOB-HMM families detected in representative strains of five *Saccharomyces* species (PDF, 52 KB)
- File S1 - Likelihood-ratio tests for variation in selection pressure along the branches of the *Saccharomyces sensu stricto* phylogeny in 5,152 orthologs (Microsoft Excel, .xls, 2.7 MB)
- File S2 - Complete lists of candidate gene gains and losses detected by computational screens in five *Saccharomyces* species (Microsoft Excel, .xls, 48 KB)
- Table S1 - Genes orthologous among representative strains of five *Saccharomyces sensu stricto* yeast species (Microsoft Excel, .xls, 2.9 MB)
- Table S3 - tRNA gene content in representative strains of five *Saccharomyces* species as detected by tRNAScan-SE (Microsoft Excel, .xls, 24 KB)
