# Supplementary material for: The Awesome Power of Yeast Evolutionary Genetics: New Genome Sequences and Strain Resources for the Saccharomyces sensu stricto Genus
Source: G3 (Bethesda). 2011 Jun 1;1(1):11–25. doi: 10.1534/g3.111.000273 (PMC3276118; doi:10.1534/g3.111.000273)
Supplement: Supporting Information [file supp_1.1.11_000273SI.pdf]

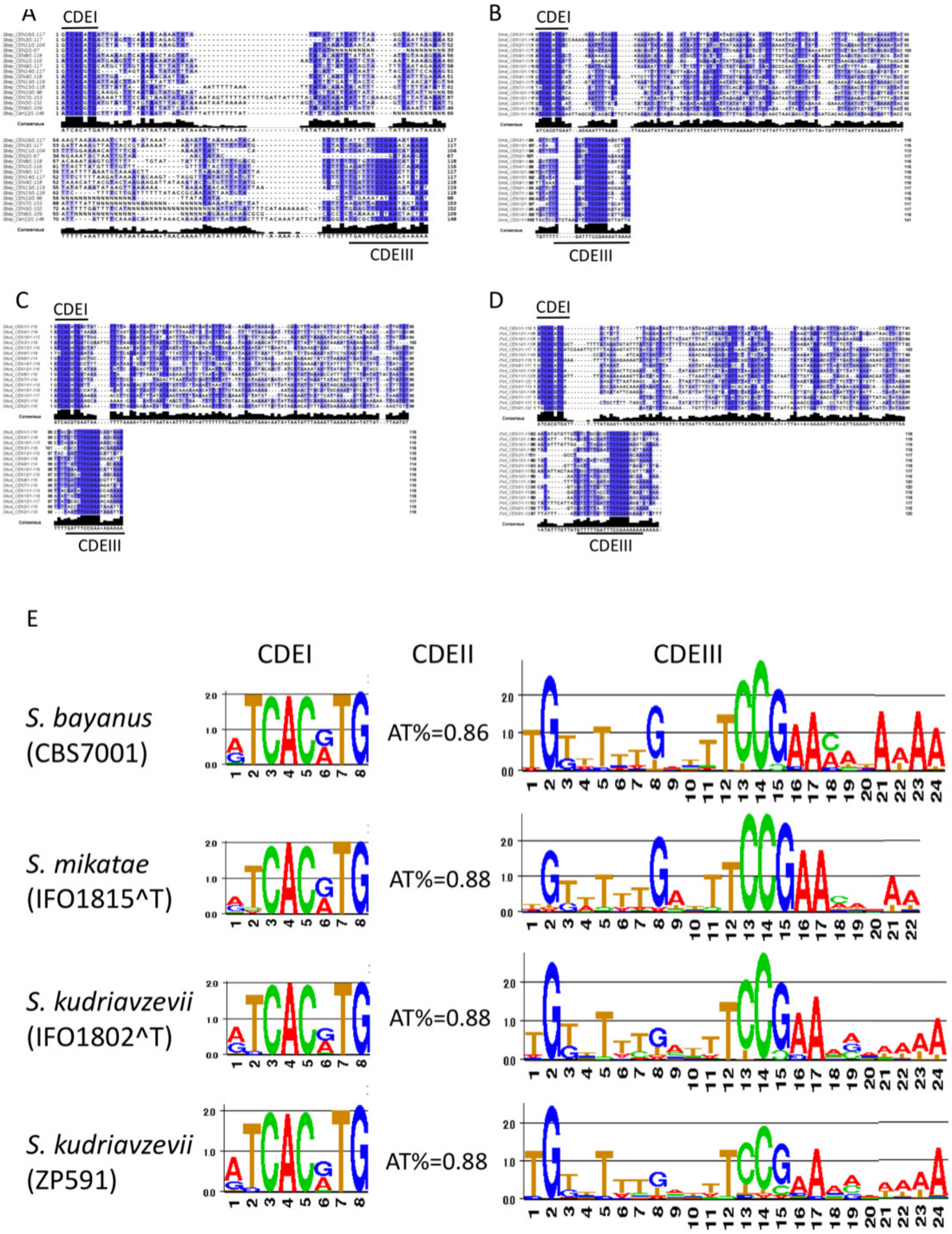

Figure S1 Multiple sequence alignment of centromeres from (A) *S. bayanus*, (B) *S. mikatae*, and *S. kudriavzevii* (C) IFO 1802T and (D) ZP 591. The alignment was generated using ClustalW-multialign (Thompson *et al.* 1994) using the default parameters for IFO 1802T, a gap extension of 0.4 for ZP 591 and *S. bayanus*, and 0.5 for *S. mikatae*. The multiple alignments were visualized using Jalview (Waterhouse *et al.* 2009). The CDEI and CDEIII domains are indicated by a black bar above the consensus sequences. (E) Weblogos (http://www.biogenio.com/logo/logo.cgi) of CDEI and CDEIII and the AT content of CDEII. The vertical axis represents the information content (measured in bits) in each alignment column. Information contents were calculated using the base frequencies in the centromere alignment for each species and thus may differ slightly from values conditioned on genomic base frequencies. Only the sequences that did not contain any gaps were used to create the logos (*e.g.* for *S. bayanus* CDEIII, only the sequence of *CEN1*, *3*, *4*, *8*, *9*, *11*, *13*, *14*, *15*, and *16* were used).

References

Thompson, J. D., D. G. Higgins and T. J. Gibson, 1994 Clustal W: improving the sensitivity of progressive multiple

sequence alignment through sequence weighting, position specific gap penalties and weight matrix choice. Nucleic Acids. Res. **22:** 4673 4680.

Waterhouse, A. M., J. B. Procter, D. M. Martin, M. Clamp and G. J. Barton, 2009 Jalview Version 2 a multiple sequence alignment editor and analysis workbench. Bioinformatics **25:** 1189 1191.

Files S1 and S2
Datasets

Files S1 and S2 are available for download as Excel files at http://www.g3journal.org/lookup/suppl/doi:10.1534/g3.111.000273/-/DC1.

File S1
Likelihood-ratio tests for variation in selection pressure along the branches of the *Saccharomyces sensu stricto* phylogeny in 5,152 orthologs. Likelihood-ratio test for positive selection in 5,152 *Saccharomyces sensu stricto* orthologs.

File S2
Complete lists of candidate gene gains and losses detected by computational screens in five *Saccharomyces* species. See Methods for details of how potential changes were identified.

Table S1 Genes orthologous among representative strains of five *Saccharomyces sensu stricto* yeast species. *S. kudriavzevii* is represented by IFO 1802T. Quality column summarizes similarity of predicted gene lengths and structures among orthologs: “length”, standard deviation(lengths)/mean(lengths) > 0.05; “structure”, number of exons/fragments differs among species; “highqual”, neither of previous two conditions met, and the orthogroup is therefore considered high-quality.

Table S1 is available for download as an Excel file at http://www.g3journal.org/lookup/suppl/doi:10.1534/g3.111.000273/-/DC1.

Table S2 YGOB-HMM families detected in representative strains of five *Saccharomyces* species. Families with two members displaying conserved synteny to the ancestral gene order (Gordon *et al.* 2009) were inferred to be descended from the ancient whole-genome duplication ("Ohnologs"; see Methods).

	YGOB-HMM Hits	YGOB-HMM Families (of 4,704)		
		Detected	Not Detected	Ohnologs
S. cerevisiae	5,490	4,605	99	527
S. paradoxus	5,440	4,547	157	542
S. mikatae	5,454	4,604	100	528
S. kudriavzevii	5,450	4,606	98	529
S. bayanus	5,559	4,611	93	522
Shared (excluding *S. paradoxus*)	-	4,589	90	520

Table S3   tRNA gene content in representative strains of five *Saccharomyces* species as detected by tRNAScan-SE. Note that a single tRNA can decode multiple codons, so the number of distinct tRNA species is fewer than 64. tRNA pseudo-genes are not reported.

Table S3 is available for download as an Excel file at http://www.g3journal.org/lookup/suppl/doi:10.1534/g3.111.000273/-/DC1.
