# Supplementary material for: The Awesome Power of Yeast Evolutionary Genetics: New Genome Sequences and Strain Resources for the Saccharomyces sensu stricto Genus
Source: G3 (Bethesda). 2011 Jun 1;1(1):11–25. doi: 10.1534/g3.111.000273 (PMC3276118; doi:10.1534/g3.111.000273)
Supplement: Supporting Information [file supp_1.1.11_FigureS1.pdf]

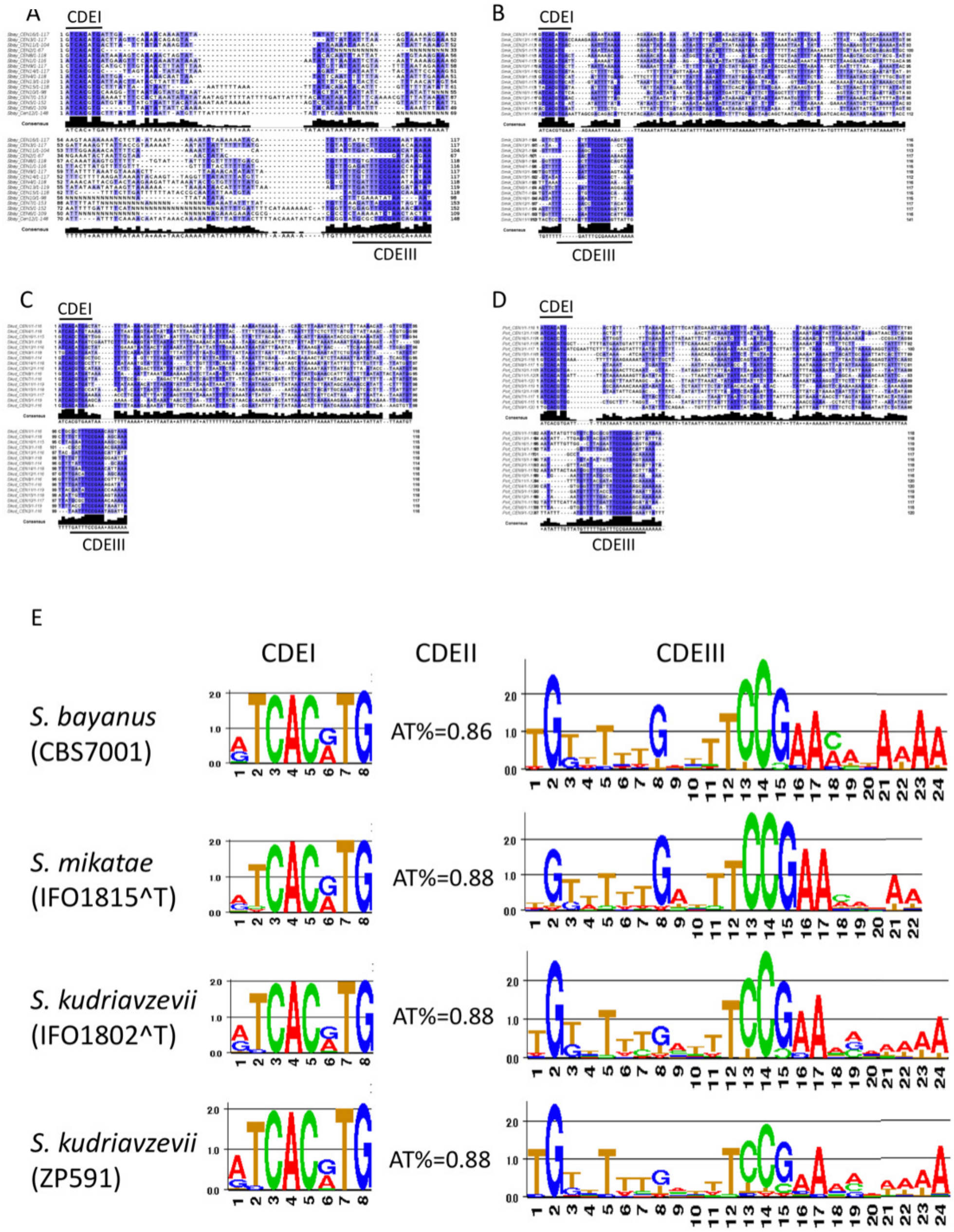

Figure S1 Multiple sequence alignment of centromeres from (A) *S. bayanus*, (B) *S. mikatae*, and *S. kudriavzevii* (C) IFO 1802T and (D) ZP 591. The alignment was generated using ClustalW-multialign (Thompson *et al.* 1994) using the default parameters for IFO 1802T, a gap extension of 0.4 for ZP 591 and *S. bayanus*, and 0.5 for *S. mikatae*. The multiple alignments were visualized using Jalview (Waterhouse *et al.* 2009). The CDEI and CDEIII domains are indicated by a black bar above the consensus sequences. (E) Weblogos (http://www.biogenio.com/logo/logo.cgi) of CDEI and CDEIII and the AT content of CDEII. The vertical axis represents the information content (measured in bits) in each alignment column. Information contents were calculated using the base frequencies in the centromere alignment for each species and thus may differ slightly from values conditioned on genomic base frequencies. Only the sequences that did not contain any gaps were used to create the logos (*e.g.* for *S. bayanus* CDEIII, only the sequence of *CEN1*, *3*, *4*, *8*, *9*, *11*, *13*, *14*, *15*, and *16* were used).

References

Thompson, J. D., D. G. Higgins and T. J. Gibson, 1994 Clustal W: improving the sensitivity of progressive multiple

sequence alignment through sequence weighting, position specific gap penalties and weight matrix choice. Nucleic Acids. Res. **22:** 4673 4680.

Waterhouse, A. M., J. B. Procter, D. M. Martin, M. Clamp and G. J. Barton, 2009 Jalview Version 2 a multiple sequence alignment editor and analysis workbench. Bioinformatics **25:** 1189 1191.
