# Supplementary material for: The Awesome Power of Yeast Evolutionary Genetics: New Genome Sequences and Strain Resources for the Saccharomyces sensu stricto Genus
Source: G3 (Bethesda). 2011 Jun 1;1(1):11–25. doi: 10.1534/g3.111.000273 (PMC3276118; doi:10.1534/g3.111.000273)
Supplement: Supporting Information [file supp_1.1.11_TableS2.pdf]

Table S2 YGOB-HMM families detected in representative strains of five *Saccharomyces* species. Families with two members displaying conserved synteny to the ancestral gene order (Gordon *et al.* 2009) were inferred to be descended from the ancient whole-genome duplication ("Ohnologs"; see Methods).

	YGOB-HMM Hits	YGOB-HMM Families (of 4,704)		
		Detected	Not Detected	Ohnologs
S. cerevisiae	5,490	4,605	99	527
S. paradoxus	5,440	4,547	157	542
S. mikatae	5,454	4,604	100	528
S. kudriavzevii	5,450	4,606	98	529
S. bayanus	5,559	4,611	93	522
Shared (excluding *S. paradoxus*)	-	4,589	90	520
